# Associations of Clinical Presentation of Coeliac Disease with Comorbidities and Complications: A Retrospective Single-Centre Analysis

**DOI:** 10.3390/jpm15020055

**Published:** 2025-01-29

**Authors:** Judit Bajor, Zsófia Vereczkei, Réka Bencs, Enikő Nagy, Míra Zsófia Peresztegi, Ivett Hegedűs, Nelli Farkas, András Tárnok, Nóra Szigeti, Zsolt Szakács

**Affiliations:** 1 First Department of Medicine, Medical School, University of Pécs, Ifjúság Str. 13, H-7624 Pécs, Hungary; szakacs.zsolt@pte.hu; 2 Institute for Translational Medicine, Medical School, University of Pécs, Szigeti Str. 12, H-7624 Pécs, Hungary; zsofia.vereczkei@etk.pte.hu; 3 Department of Sport Nutrition and Hydration, Institute of Nutritional Science and Dietetics, Faculty of Health Sciences, University of Pécs, Vörösmarty Mihály Str. 4, H-7621 Pécs, Hungary; 4 Second Department of Internal Medicine and Nephrological Centre, Medical School, University of Pécs, Pacsirta Str. 1, H-7624 Pécs, Hungary; bencs.reka@pte.hu (R.B.); szigeti.nora@pte.hu (N.S.); 5 Department of Emergency Medicine, Medical School, University of Pécs, Ifjúság Str. 13, H-7624 Pécs, Hungary; eniko.nagy@pte.hu; 6 Medical School, University of Pécs, Szigeti Str. 12, H-7624 Pécs, Hungary; peresztegi.mira@pte.hu; 7 Department of Pathology, Medical School, University of Pécs, Szigeti Str. 12, H-7624 Pécs, Hungary; hegedus.ivett@pte.hu; 8 Institute of Bioanalysis, Medical School, University of Pécs, Szigeti Str. 12, H-7624 Pécs, Hungary; nelli.farkas@aok.pte.hu; 9 Department of Paediatrics, Medical School, University of Pécs, József Attila Str. 7, H-7623 Pécs, Hungary; tarnok.andras@pte.hu

**Keywords:** coeliac disease, clinical presentation, comorbidities, complications

## Abstract

**Background:** The clinical presentation of coeliac disease (CD) is various and may influence disease course. We aimed to investigate the associations of clinical presentation with comorbidities and disease complications in a cohort of Hungarian coeliac patients. **Methods:** In this retrospective study, data of consecutive CD patients were analysed. Clinical presentation (classical vs. non-classical), extraintestinal manifestations and comorbidities (anaemia, metabolic bone disease, dermatitis herpetiformis, IgA deficiency, chromosomal abnormalities, autoimmune diseases and malignancy) were assessed. Student’s *t*-test (for age at diagnosis) and the Chi-squared test or Fisher’s exact test (for categorical variables) were applied as analyses. **Results:** A total of 738 patients were included. In classical vs. non-classical comparisons, classical presentation was significantly associated with metabolic bone disease (59 vs. 36%, respectively, *p* < 0.001), anaemia (47 vs. 38%, respectively, *p* = 0.027) and malignancy (6 vs. 2%, respectively, *p* = 0.006); however, autoimmune diseases and dermatitis herpetiformis were more common with non-classical presentation (23 vs. 31%, *p* = 0.02, and 5 vs. 16%, *p* = 0.014, respectively). **Conclusions:** Our findings confirm that clinical presentation is associated with certain comorbidities and complications in CD. More personalised follow-up may be recommended based on clinical presentation.

## 1. Introduction

Coeliac disease (CD) is one of the most common immune-mediated disorders, which develops as a response to gluten among genetically predisposed individuals. The overall worldwide disease prevalence is currently about 1%, yet it shows an increasing tendency, likely due to improving testing methodology and rising disease awareness among healthcare providers [1]. Historically, CD has been characterised by malabsorption, including diarrhoea, weight loss, failure to thrive and various nutritional deficiencies. However, these cases represent only the ‘tip of the iceberg’ because many patients are diagnosed based on atypical symptoms, extraintestinal manifestations, associated immune-mediated diseases or are detected by serological screening while being asymptomatic. The active case-finding strategy recommended by the guidelines might miss some cases due to the high proportion of subclinical presentation [1,2]. Diagnostics, treatment and follow-up are managed according to well-defined, uniform principles, regardless of whether the initial clinical symptoms were classical, non-classical or asymptomatic [3,4,5]. In addition to maintaining adherence to a gluten-free diet (GFD), the biggest challenge in patient care is currently the management of comorbidities and complications. There is no evidence on to what extent clinical symptoms at the time of the diagnosis determine the further fate of the patient. One might reasonably assume that the more severe, classical symptoms are associated with more severe intestinal histological damage and a higher anti-tissue transglutaminase (tTG) antibody titre, and that these patients are potential candidates for more serious outcomes during the disease course [6,7]. However, total villous atrophy and a high antibody titre may be present in an asymptomatic patient, which questions a direct correlation between clinical presentation and histological severity [8].

The question arises as to whether the assessment of clinical presentation at the time of diagnosis helps to determine which patients should be followed up more closely and which are at a higher risk of developing complications. Concerning this, data are scant and the literature is inconsistent. In a paediatric study, the extent of villous atrophy did not correlate with clinical severity, but long-term complications or comorbidities did so. [9]. The results of a large multi-centre study indicate that being diagnosed at a late age and having a classical clinical presentation phenotype are predictive of the development of severe complications later in life, which emphasises the need for personalised care and follow-up [10].

This study aimed to investigate the associations of clinical presentation with comorbidities and disease complications in a cohort of Hungarian coeliac patients.

## 2. Materials and Methods

The study is reported in conformity with the STROBE Statement [11]. The study was conducted in accordance with the Declaration of Helsinki and approved by the Regional and Local Research Ethics Committee of University Pécs, Pécs, Hungary (ref.no 6918).

### 2.1. Study Design and Data Source

The University of Pécs is the regional referral centre for coeliac patients throughout southwest Hungary, Baranya County. In this retrospective cohort study, data collection was performed using the medical record database of the University of Pécs (eMedSolution, T-Systems Hungary Ltd., Budapest, Hungary, Version: 2023/Q1/1 (20230127151442)).

### 2.2. Data Collections, Study Population and Definition of Study Variables

In our analysis, we searched the electronic database between 1 January 2007 and 31 December 2019. We identified all coeliac patients who attended the clinics in this period using the International Classification of Diseases, 10th Revision coding of CD (ICD-1 K90.0). All patients (without age restriction) registered at any time with a primary or secondary diagnosis of CD were included in this study. Social Security Numbers were used to identify duplicate records. The diagnosis of CD was reassessed by checking the original data on CD-specific serology and intestinal histology according to the valid guidelines in the year of the diagnosis. The incorrect or uncertain cases were excluded. Only the patients with a well-definable clinical phenotype were included in the study. The collected data were filled into the pre-defined data collection table. A total of five investigators were involved in the acquisition of data.

This study population (or a part of it) has been analysed in previous works of our study group, yet with different clinical questions [12,13].

Patients’ data were systematically collected. Information referencing the gender, age at diagnosis, clinical phenotype, diagnostic histology, serology, extraintestinal manifestation (anaemia, metabolic bone disease), coexistent immune-mediated diseases (dermatitis herpetiformis (DH), autoimmune diseases (AD)), chromosomal abnormality and malignancy were obtained manually and stored in a database.

Regarding the clinical presentation at diagnosis, we divided this into classical and non-classical as per the Oslo criteria; the classical presentation was defined based on the presence of signs and symptoms of malabsorption, diarrhoea, weight loss and failure to thrive [14]. Silent cases were included in the non-classical group. Diagnostic histological samples were described at the time of the diagnosis by a gastrointestinal histopathologist using the modified Marsh classification [15]. Commercially available ELISA kits (Orgentec Diagnostika GmbH, Mainz, Germany) for the assay of tTG antibodies were used. A tTG level > 10 U/mL was considered positive. A high tTG level was defined as being greater than 10 times the upper limit of normal (ULN). In seronegative cases, the diagnosis was made based on histology. Haemoglobin levels < 130 g/L and < 120 g/L indicated anaemia in males and females, respectively. Metabolic bone disease (including osteopenia and osteoporosis) was defined by measuring a T-score < −1.0 or < −2.0 standard deviation by dual-energy X-ray absorptiometry (DEXA). IgA deficiency was defined as a condition in which total serum IgA level, measured by nephelometry, was below 0.07 g/L. Concurrent AD, malignancies and DH and chromosomal abnormality (Down’s or Turner syndrome) were assessed as well.

### 2.3. Statistical Analysis

In descriptive statistics, age at diagnosis was handled as a continuous variable and the mean with standard deviation was calculated. Categorical variables were described with absolute counts and relative frequencies (%). To examine the association between clinical presentation and other variables through comparative analysis, Student’s *t*-test (for age at diagnosis) and the Chi-squared test or Fisher’s exact test (for categorical variables) were applied. *p* ˂ 0.05 indicated statistical significance. All calculations were made using IBM-SPSS ver. 28 (IBM Corp. Released 2021. IBM SPSS Statistics for Windows, Version 28.0. Armonk, NY, USA: IBM Corp).

## 3. Results

### 3.1. Patients’ Characteristics

**Figure 1:**
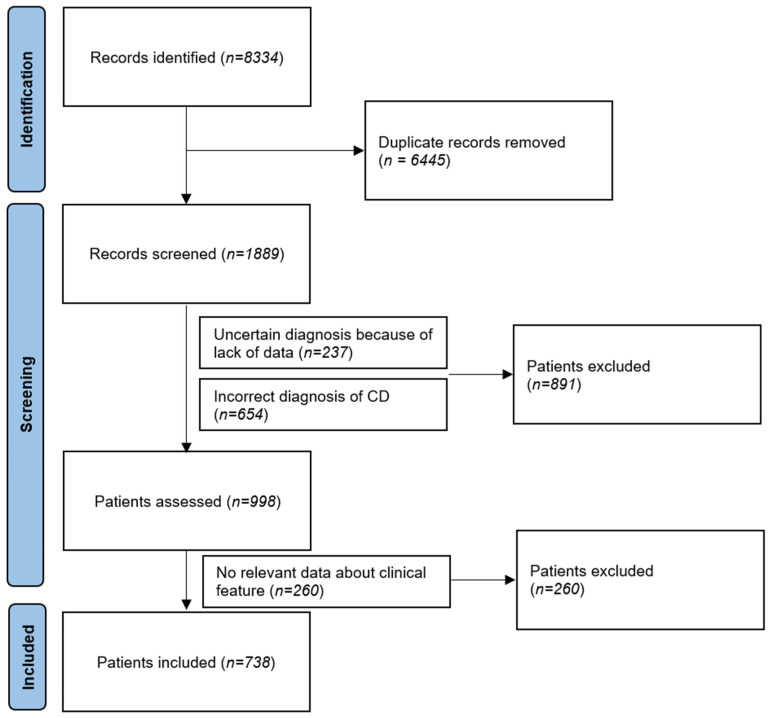
Flowchart of study.

As a result of searching the database, we acquired 8334 cases, from which a balance of 1889 remained after excluding duplicate records. In the reassessment of the search yield, 654 cases of coeliac disease were excluded from the study (usually due to miscoding or misdiagnosis) and further 237 cases could not be evaluated for the study due to uncertain diagnosis. Among 260 patients, the classification of clinical presentation was ambiguous, so they were also excluded from the study. The flowchart of the search and selection is presented in Figure 1.

A total of 738 patients were included in the analysis. Patients’ characteristics are summarised in Table 1. Approximately one-fourth of the patients were males. Out of the 738 patients, 290 (39%) had classical and 448 (61%) patients had non-classical CD (55 silent cases).

### 3.2. Association of Clinical Presentation with Metabolic Bone Disease, Anaemia and IgA Deficiency

Table 2 summarises the data of comorbidities and complications. Metabolic bone disease and anaemia were more common with classical presentation (*p <* 0.001 and *p* = 0.027, respectively). The proportion of IgA deficiency did not significantly differ between the groups (*p* = 0.089).

### 3.3. Association of Clinical Presentation with Immune-Mediated Comorbidities

A total of 207 patients (28%) were affected by another AD, of which 164 had 1 (22.2%) and 43 had more than 1 (5.8%) ADs. The most frequent AD was thyroid disease, which occurred in 12.3% of all patients and accounted for 44.3% of all ADs. The prevalence of AD was found to be significantly higher among CD patients with a non-classical presentation compared to those with a classical presentation (*p* = 0.025) (Table 2). DH occurred in 61 patients (8.3%); however, its frequency was significantly higher with non-classical presentation (*p* = 0.014). The type and frequency of immune-mediated diseases are presented in Table 3. The clinical characteristics of CD patients with multiplex ADs are presented in Table S1.

### 3.4. Association of Clinical Presentation with Chromosomal Abnormalities

In our cohort of patients, ten had Down’s syndrome (1.4%) and six had Turner syndrome (0.8%). All patients with Turner syndrome and 60% of the patients with Down’s syndrome had non-classical presentations, but the difference between groups was non-significant (*p* = 0.237) (Table 2).

### 3.5. Association of Clinical Presentation with Malignancies

Malignancy was diagnosed in a total of 23 patients (3.1%) (the male-to-female ratio was 8 to 15). The mean age upon CD diagnosis in patients with malignancy was 34.9 years (vs. 22.8 in those without malignancy). The mean age at diagnosis of malignancy was 43.0 years (five cases were under 18 years). Malignancy was diagnosed prior to the CD diagnosis in three cases, and simultaneously in three cases. The type of malignancy and characteristics of the patients with malignancy are summarised in Table S2. Classical clinical presentation was significantly associated with malignancies (*p* = 0.006) (Table 2).

## 4. Discussion

Our study aimed to find associations of clinical presentation with comorbidities and disease complications in CD. We analysed data from a cohort of CD patients who attended our hospitals at the University of Pécs (Pécs, Hungary) between 2007 and 2019.

The number of CD patients has grown significantly in recent decades. This tendency has been observed worldwide [16,17,18,19]; yet, some studies have reported a stagnation in the growth rate [20,21]. A true increase in incidence, better awareness and efficacy of screening programmes are behind this phenomenon [22,23,24]. In our case, many factors including the introduction of modern serology methods, standardisation of patient care, the establishment of a Coeliac Centre, the active case-finding strategy, cooperation with other specialists and propagation of family screening have led to a more efficient diagnostic efficacy. We observed a marked shift in clinical presentation in the last several decades: non-classical CD became significantly more prevalent [12]. This change in phenotype was also observed in many other studies in children [25,26,27] and adults [24,28,29,30,31]. The mean age at diagnosis of CD was quite low in our mixed-age (children–adults) study population, as nearly half of the patients were diagnosed in childhood. Surprisingly, our patients with classical CD were slightly older than those with the non-classical phenotype. This is not in line with the literature’s data, which, rather, shows that the dominance of classical CD is limited to children under 3 years of age [32,33] and to some countries beyond Europe [34,35]. In our cohort of patients, female predominance is also remarkably prominent (73.7%), which is in line with international trends [19].

The analysis of the comorbidities led to various results. There are little data regarding chromosomal abnormalities (Down’s syndrome, Turner syndrome) in CD; however, these conditions are more common among coeliac patients than in the average population (the prevalence of Down’s syndrome is about 1.4%, that of Turner syndrome is 0.26% in CD). Therefore, screening for CD is highly recommended in this population [36,37,38]. In our study, Down’s syndrome was associated mostly with non-classical presentation. In patients with Turner syndrome, CD manifested exclusively with non-classical presentation. Even so, we could not establish a significant difference between the groups due to the small number of cases. This needs further investigation, but this experience underlines the importance of screening of CD in these groups, even in the absence of typical symptoms.

Regarding IgA deficiency, we failed to reveal an association with clinical presentation.

Extraintestinal manifestations are common in patients with CD, affecting many organs and organ systems [39,40]. It is not clear what factors predispose their development; however, it is advisable to identify and treat them as soon as possible. It is not known whether they have a prognostic role in the disease course. It is reasonable to think that patients with more severe symptoms and generalised malabsorption are expected to have more complications. This is supported by the study of Nurminen et al. reporting more frequent complications among patients who developed more severe clinical symptoms and histology [41].

According to a recently published reviews, the frequency of anaemia varies in CD (ranging from 12% up to 85%) [42,43]. The prevalence of anaemia in our patient population is high (41%), which underlines the importance of routine monitoring of the anaemia-related parameters (vitamin levels, iron homeostasis-related parameters) upon diagnosis and during the GFD. The cause of anaemia in CD is complex, but malabsorption logically has a pivotal role [44]. Results of a Finnish study reporting that anaemia is more frequent in cases with severe symptoms corroborate this hypothesis [45].

Metabolic bone disease also occurs in a high percentage of our cohort (47.5%), more commonly than in other reports. These findings highlight the need of osteodensitometry at the time of diagnosis, and also to treat the metabolic bone disorder and to monitor the effectiveness of the therapy [29,46,47]. The results of our study suggest that bone metabolism disorders are more likely to develop among patients with classical symptoms; therefore, these patients require special attention. This conclusion is not surprising because bone metabolism disorders are likely due to the generalised malabsorption typically occurring in classical CD.

The prevalence of ADs among individuals with CD is nearly three times higher than that expected in the general population, with 20–30% of CD patients having at least one AD [19,48]. With a worldwide increasing trend in the rate of ADs, it imposes a real burden on society [18]. We detected ADs in 28% of patients, which is comparable with data reported from other centres [28,29,46,49,50,51]. Among our patients, the most common AD was autoimmune thyroiditis, with a similar rate to that previously published in a Hungarian university clinic study [50,52]. The prevalence of DH was also similar to that previously reported [49,50,52].

In our study, non-classical clinical presentation was associated with the development of ADs. In another study, it was also observed that family history of AD, being overweight upon diagnosis and delay of diagnosis were associated with an increased risk of the development of another AD [49]. However, in other reports, gender, coeliac symptoms, serology titre, HLA type and histopathological stage had no predictive role for the coexistence of AD among patients with CD [53,54].

The role of age upon diagnosis in the development of AD is deemed controversial. In addition, is it not obvious that early diagnosis of CD prevents the development of ADs [55,56]. The most common belief says that gluten exposure predisposes to autoimmunity and most ADs improve with a GFD [39,57,58]. This hypothesis supports the benefit of early diagnosis. Our study suggests that ADs should be expected to occur among individuals with non-classical symptoms.

Patients affected by CD have a higher risk of developing tumours, confirmed by a recent Swedish study encompassing a large patient population [59]. Although the risk is mitigated during a GFD, it remains high regardless of mucosa healing, which does not significantly modify the risk [60,61]. GFD seems to work against the development of lymphomas and small bowel tumours, yet, some other types of tumours (colon, breast) occur less frequently in untreated CD patients [62]. Diagnosis at a higher age (the role of chronic inflammation) and male gender can also be prognostic factors for malignancy [29].

The scientific literature on the role of clinical presentation on tumour risk is controversial. In Rampertab’s study, clinical presentation (classical or not) was not proven to be a predictor of tumour development [30]. In contrast, in a large Italian study consisting of 2225 (adult CD) patients with classical presentation, the incidence of complicated CD (refractory CD, lymphoma, small bowel tumour) was seven times higher with classical presentation [10]. Complications were associated with an early age upon diagnosis and classical clinical presentation. In our study, the classical clinical presentation was also associated with tumours. The CD-specific types of tumours (small bowel tumour and enteropathy-associated T-cell lymphoma) did not occur. However, a few rare tumour types (embryonal testicular carcinoma, testicular teratocarcinoma, osteogenic sarcoma, atypical teratoid rhabdoid brain tumour) were also observed, usually developing at an early age, prior to the diagnosis of CD. These patients had predominantly non-classical presentation. In contrast, patients with a classical presentation were characterised by tumour types being not common among the general population. This seems to be in contradiction with the scientific literature, where it is believed that untreated CD may be protective due to the impaired absorption of carcinogens [63].

There are many challenges during the follow-up of patients with CD. Currently, the main purpose of the therapy is to maintain and adhere to a GFD and to monitor antibody titres. With a well-managed diet, in most cases, the symptoms disappear, the mucosa regenerates, coeliac-specific antibodies normalise and deficiency states resolve. Our study shows that up to more than 40% of patients had comorbidities or complications that require an intervention from the treating physician (e.g., management of anaemia, bone diseases and ADs). In some patients, these comorbidities, especially ADs, may appear during the follow-up, even years or decades after CD diagnosis. These conditions should be actively searched for, e.g., by regularly monitoring the patient’s thyroid function. Clinical guidelines make recommendations for monitoring, yet now, the optimal follow-up strategy is unclear and there is no consensus [1,3,4]. The guidelines recommend that strict adherence to a GFD is important to prevent complications; however, there are no differences in the recommendations for therapy and follow-up based on the clinical presentation (whether malabsorption is present at diagnosis or not). Only the British guideline mentions that “symptomatic patients should be evaluated more thoroughly than asymptomatic” [64]. Our results suggest that there is a need for the focus of patient care needs to become slightly different: a personalised management approach is likely to be more beneficial. We highly agree with Dr Biagi and colleagues’ proposal stating that personalised follow-up based on clinical parameters already available at the time of the diagnosis is recommended (to fit a model to assess the combined prognostic role of age at diagnosis and clinical type of CD) [10].

The strength of this study is the size of the population and its coverage (the study reports data of all coeliac patients of one of the four university centres of Hungary). To date, this is the largest study describing data referencing Hungarian coeliac patients in such vivid clinical detail. A descriptive analysis of 178 CD patients of another Hungarian university centre was published earlier [50,52]. We analysed CD patients at any age: both children and adults were included. Systematic patient selection and consecutive inclusion of all appropriate patients mitigated the selection bias. Internationally, relatively few large studies are available describing the clinical presentation of CD. In the literature, the association between clinical phenotype and other clinical parameters has already been analysed; however, only in a few instances [7,10].

A limitation of this study is, obviously, its retrospective nature. Missing data were common prior to 2007 because the electronic clinical databases were harmonised in this year. Where the original data (histological or serological tests, osteodensitometry results, haemoglobin level, etc.) were not available, an analysis could not be performed. A further limitation was that we did not investigate the chronological relation across the diseases. We have not evaluated GFD adherence, which may also influence the incidence of complications.

## 5. Conclusions

Our study shows that classical presentation upon diagnosis is associated with more cases of metabolic bone disease, anaemia and malignancies compared to non-classical presentation. However, non-classical presentation was associated with more immune-mediated comorbidities (DH and AD) compared to classical presentation. These imply that CD patients may benefit from a more personalised follow-up tailored by clinical presentation upon diagnosis and highlight the importance of the management of comorbid conditions in CD. Clinicians providing care for CD patients should be aware of comorbidities and disease complications and should perform regular screening accordingly. Our findings should be validated in prospective cohort studies.

## Figures and Tables

**Table 1:** Characteristics of patients included.

	Number of Cases with Available Data	Total Cohort of Patients	Patients with Classical Presentation	Patients with Non- Classical Presentation
Age at diagnosis (mean, standard deviation)	738	22.8 ± 17.1	24.4 ± 18.8	21.8 ± 15.9
Age at diagnosis				
<18 years	362	362 (49%)	122 (34%)	240 (66%)
≥18 years	376	376 (51%)	168 (45%)	208 (55%)
Sex (*n*)	738	738	290 (39%)	448 (61%)
Male		194 (26%)	67 (35%)	127 (65%)
Female		544 (74%)	223 (41%)	321(59%)
Diagnostic histology (*n*)	462			
Marsh 1		6 (1%)	2 (33%)	4 (67%)
Marsh 2		11 (2%)	4 (36%)	7 (64%)
Marsh 3a		61 (13%)	22 (36%)	39 (64%)
Marsh 3b		126 (27%)	50 (40%)	76 (60%)
Marsh 3c		258 (56%)	103 (40%)	155 (60%)
Diagnostic tTG serology (*n*)	566			
tTG IgA positive low titre		159 (28%)	53 (33%)	106 (67%)
tTG IgA positive high titre		407 (72%)	139 (34%)	268 (66%)

**Table 2:** Association of clinical presentation with comorbidities and disease complications.

	Number of Cases with Available Data	Total Cohort of Patients	Patients with Classical Presentation	Patients with Non-Classical Presentation	*p*-Value (Classical vs. Non-Classical Presentation)
IgA deficiency (*n*)	318	36/318 (11%) **	18/118 (15%)	18/200 (9%)	0.089
Dermatitis herpetiformis (*n*)	738	61/738 (8%)	15/290 (5%)	46/448 (16%)	0.014 *
Anaemia (*n*)	656	272/656 (41%)	121/259 (47%)	151/397 (38%)	0.027 *
Metabolic bone disease (*n*)	244	116/244 (48%)	71/120 (59%)	45/124 (36%)	<0.001 *
osteoporosis		63/244 (26%)	46/120 (38%)	17/124 (14%)	
osteopenia		53/244 (22%)	25/120 (21%)	28/124 (23%)	
Autoimmune diseases (*n*)	738	207/738 (28%)	68/290 (23%)	139/448 (31%)	0.025 *
one		164/207 (79%)	55/290 (19%)	109/448 (24%)	
more than one		43/207 (21%)	13/290 (5%)	30/448 (7%)	
Chromosomal abnormality (*n*)	738	16/738 (2%)	4/290 (1%)	12/448 (3%)	0.237
Down’s syndrome		10/738 (1%)	4/290 (1%)	6/448 (2%)	
Turner syndrome		6/738 (1%)	0/290 (0%)	6/448 (2%)	
Malignancy (*n*)	738	23/738 (3%)	16/290 (6%)	7/448 (2%)	0.006 *

* indicates statistical significance (*p* < 0.05); ** indicates percentages are rounded to the nearest whole number.

**Table 3:** Immune-mediated diseases in patients with CD.

Immune-Mediated Diseases	Number of Patients	% of Cohort (*n* = 738)
Autoimmune thyroid disease	91	12.33%
Dermatitis herpetiformis	61	8.26%
Type 1 diabetes mellitus	52	7.04%
Raynaud syndrome	30	4.06%
IBD (11 UC, 4 Crohn’s disease, 1 indeterminate)	16	2.16%
Sjögren’s disease	11	1.49%
Autoimmune liver diseases (4 AIH, 5 PBC, 2 PSC)	11	1.49%
Psoriasis	7	0.94%
Systemic lupus erythematosus	5	0.67%
Alopecia areata	5	0.67%
Rheumatoid arthritis (RA 1, JIA 2, seronegative RA 1)	4	0.54%
Vitiligo	4	0.54%
Sarcoidosis	3	0.40%
Antiphospholipid syndrome	3	0.40%
Immune thrombocytopenic purpura	2	0.27%
Dermatomyositis	2	0.27%
Lichen ruber planus	2	0.27%
MCTD	2	0.27%
Pulmonary fibrosis	2	0.27%
Scleroderma	2	0.27%
IgA nephropathy	1	0.13%
Lichen oris	1	0.13%
Myasthenia gravis	1	0.13%
Vasculitis	1	0.13%
Polymyositis	1	0.13%
Autoimmune haemolytic anaemia	1	0.13%
Multiple sclerosis	1	0.13%

IBD: inflammatory bowel disease; UC: ulcerative colitis; RA: rheumatoid arthritis; JIA: juvenile idiopathic arthritis; AIH: autoimmune hepatitis; PBC: primary biliary cholangitis; PSC: primary sclerotizing cholangitis; MCTD: mixed connective tissue disease.

## Data Availability

The original contributions presented in this study are included in the article/Supplementary Material. Further inquiries can be directed to the corresponding author. The raw data supporting the conclusions of this article will be made available by the authors on request.

## Supplementary Materials

The following supporting information can be downloaded at: https://www.mdpi.com/article/10.3390/jpm15020055/s1, Table S1: Clinical characteristics of CD patients with multiplex autoimmune diseases; Table S2: Clinical characteristics of patients with tumours.
