# Supplementary material for: Associations of Clinical Presentation of Coeliac Disease with Comorbidities and Complications: A Retrospective Single-Centre Analysis
Source: J Pers Med. 2025 Jan 29;15(2):55. doi: 10.3390/jpm15020055 (PMC11856780; doi:10.3390/jpm15020055)
Supplement: Supplementary file 1 [file jpm-15-00055-s001.zip › jpm-3366252-supplementary.pdf]

Table S1. Clinical characteristics of CD patients with multiplex autoimmune diseases.

Gender	Age at diagnosis	Clinical presentation	Autoimmune diseases
female	24	non-classical	Sjögren's disease, SLE, ITP, autoimmune thyroiditis
male	13	non-classical	Sjögren's disease, SLE
female	62	non-classical	Sjögren's disease, PBC, dermatitis herpetiformis
female	28	non-classical	autoimmune hepatitis, Sjögren's disease, SLE
male	39	non-classical	autoimmune hepatitis, Sjögren's disease, PBC
female	26	non-classical	alopecia areata, RA
female	24	non-classical	alopecia areata, autoimmune thyroiditis
female	48	classical	Raynaud syndrome, Sjögren's disease, RA, autoimmune thyroiditis
female	41	non-classical	Raynaud syndrome, Sjögren's disease, autoimmune thyroiditis
female	45	non-classical	Raynaud syndrome, Sjögren's disease, autoimmune thyroiditis
female	34	non-classical	Raynaud, Sjögren's disease
female	31	classical	Raynaud, PBC
female	6	non-classical	Raynaud, ulcerative colitis, PSC
male	44	non-classical	Raynaud, ulcerative colitis
female	18	classical	Raynaud, SSc
male	54	non-classical	Raynaud, MCTD, pulmonary fibrosis, polymyositis
female	29	classical	Raynaud, autoimmune thyroiditis
female	69	non-classical	Raynaud, autoimmune thyroiditis
female	27	classical	Raynaud, autoimmune thyroiditis
female	5	classical	Raynaud, autoimmune thyroiditis
female	30	non-classical	Raynaud, RA
male	23	non-classical	Raynaud, psoriasis, antiphospholipid syndrome
female	34	non-classical	Raynaud, psoriasis, autoimmune thyroiditis, antiphospholipid syndrome
female	51	classical	IgA nephropathy, autoimmune thyroiditis
female	45	classical	IgA nephropathy, autoimmune thyroiditis
female	32	classical	IDDM, Raynaud
female	16	non-classical	IDDM, autoimmune thyroiditis
female	32	non-classical	IDDM, autoimmune thyroiditis
male	9	non-classical	IDDM, autoimmune thyroiditis
male	3	non-classical	IDDM, autoimmune thyroiditis

female	6	non-classical	IDDM, autoimmune thyroiditis
female	11	non-classical	IDDM, autoimmune thyroiditis
male	17	non-classical	IDDM, autoimmune thyroiditis
female	6	non-classical	IDDM, autoimmune thyroiditis
female	5	non-classical	IDDM, autoimmune thyroiditis
female	17	non-classical	IDDM, autoimmune thyroiditis
male	11	non-classical	IDDM, dermatomyositis
female	50	non-classical	psoriasis, lichen ruber
female	15	non-classic	vitiligo, autoimmune thyroiditis
female	1	classical	vitiligo, autoimmune thyroiditis
female	3	classical	vitiligo, autoimmune thyroiditis
female	36	classical	Raynaud, psoriasis
female	51	classical	sarcoidosis, lichen oris

SLE: systemic lupus erythematosus, ITP: idiopathic thrombocytopenic purpura, PBC: primary biliary cholangitis, PSC: primary sclerotizing cholangitis, RA: rheumatoid arthritis, IDDM: insulin dependent diabetes mellitus, IgA: Immunoglobulin A.

Table S2. Clinical characteristics of patients with tumours.

Gender	Age at CD diagnosis	Age at tumour diagnosis	Year of the birth	Year of CD diagnosis	Clinical feature	Type of malignancy
female	29	36	1980	2009	classical	mamma carcinoma
male	46	55	1959	2005	classical	lung carcinoma
female	5	62	1954	1959	classical	endometrial carcinoma
male	42	48	1952	1994	classical	colorectal carcinoma
female	59	75	1941	2000	classical	pancreas carcinoma
male	18	13	1995	2013	classical	testicular teratocarcinoma
male	23	16	1993	2016	non-classical	embryonal testis carcinoma
male	28	61	1953	1981	classical	colorectal carcinoma
female	55	55	1954	2009	classical	colorectal carcinoma
female	69	73	1945	2014	non-classical	mucinous ovarian tumour
female	36	38	1972	2008	classical	malignant melanoma

male	3	6	1995	1998	non-classical	NHL
female	23	15	1989	2012	non-classical	osteogenic sarcoma
male	5	5	2009	2014	non-classical	atypical teratoid rhabdoid brain tumour
female	47	35	1969	2016	classical	colorectal carcinoma
male	1	50	1968	1969	classical	soft tissue sarcoma
female	48	51	1959	2007	classical	mamma carcinoma
female	57	81	1932	1989	classical	colorectal carcinoma
female	41	45	1967	2008	classical	renal cell carcinoma
female	10	15	1997	2007	non-classical	thyroid papillary carcinoma
female	42	24	1969	2011	classical	malignant melanoma
female	64	64	1952	2016	non-classical	meningioma
female	52	71	1934	1986	classical	NHL

NHL: Non-Hodgkin Lymphoma
